# Understanding the role of video direct observed therapy for patients on an oral short-course regimen for multi-drug resistant tuberculosis: findings from a qualitative study in Eswatini

**DOI:** 10.1186/s12879-024-09744-9

**Published:** 2024-08-15

**Authors:** Esther Mukooza, Bernadette Schausberger, Nqobile Mmema, Velibanti Dlamini, Aung Aung, Bernhard Kerschberger, Iza Ciglenecki, Debrah Vambe, Alison Wringe

**Affiliations:** 1 Médecins sans Frontières (OCG), P.O.Box 325, Mantsholo Road, Mbabane, Eswatini; 2 Médecins sans Frontières (OCG), Geneva, Switzerland; 3 National TB Control Programme (NTCP), Manzini, Eswatini; 4 London School of Hygiene and Tropical Medicine (LSHTM), London, UK

**Keywords:** MDR-TB, Eswatini, Qualitative, vDOT, Oral short course treatment

## Abstract

**Background:**

Improving treatment success rates among multi drug-resistant tuberculosis (MDR-TB) patients is critical to reducing its incidence and mortality, but adherence poses an important challenge. Video-based direct observed therapy (vDOT) may provide adherence benefits, while addressing the time and cost burden associated with community treatment supporter (CTS)-DOT. This study explored experiences of patients, family members and healthcare workers with different DOT modalities for adherence support in Eswatini.

**Methods:**

Between April 2021 and May 2022, thirteen men and five women with MDR-TB, ten healthcare workers, and nine caregivers were purposively sampled to include a range of characteristics and experiences with DOT modalities. Data were generated through individual in-depth interviews and a smartphone messaging application (WhatsApp). Data coding was undertaken iteratively, and thematic analysis undertaken, supported by Nvivo.

**Results:**

Four themes emerged that reflected participants’ experiences with different DOT modalities, including stigma, efficiency, perceived risks of TB acquisition, and patient autonomy. vDOT was appreciated by patients for providing them with privacy and shielding them from stigmatisation associated with being seen in TB clinics or with community treatment supporters. vDOT was also seen as more efficient than CTS-DOT. Health workers acknowledged that it saved time, allowing them to attend to more patients, while many patients found vDOT more convenient and less expensive by removing the need to travel for in-person consultations. Health workers also appreciated vDOT because it reduced risks of TB acquisition by minimising exposure through virtual patient monitoring. Although many patients appreciated greater autonomy in managing their illness through vDOT, others preferred human contact or struggled with making video recordings. Most family members appreciated vDOT, although some resented feeling removed from the process of supporting loved ones.

**Conclusions:**

vDOT was generally appreciated by MDR-TB patients, their family members and health workers as it addressed barriers to adherence which could contribute to improved treatment completion rates and reduced workplace exposure. However, patients should be offered an alternative to vDOT such as CTS-DOT if this modality does not suit their circumstances or preferences.

**Supplementary Information:**

The online version contains supplementary material available at 10.1186/s12879-024-09744-9.

## Background

The multidrug-resistant and rifampicin-resistant tuberculosis (MDR and RR-TB) epidemic is a growing public health problem, with a 3.1% increase in incidence documented between 2020 and 2021, leading to 450,000 new cases globally [1]. In Eswatini, where the dual HIV and TB epidemics have had devastating social and economic impacts, the total annual number of notified MDR-TB cases stood at 73 in 2022, up from 60 in 2020, following a previously substantial decline from 642 in 2012 [2]. In addition to inadequate notification rates, progress is limited by sub-optimal MDR-TB treatment outcomes which can further onward transmission. Despite steady improvements since 2015, the treatment success rate among patients initiating MDR-TB treatment in 2020 in Eswatini was still under 80% [2, 3]. Multiple challenges undermine the ability of patients with MDR-TB to complete their treatment, including drug side-effects, stigma, and long distances to health facilities for observation of daily treatment-taking [4].

Direct observed therapy (DOT) remains a cornerstone of TB treatment, with the World Health Organisation (WHO) recommending that it be administered by trained lay providers or healthcare workers over administration by family members or unsupervised treatment, ideally in the community or home [5]. Several studies have shown that community-based and facility-based DOT enables supportive relationships to be developed between patients and providers which can improve treatment adherence and completion. However, there are also many challenges with the implementation of DOT, including its cost and time-consuming nature for both provider and patient, as well as issues with stigmatisation arising from the lack of privacy in treatment-taking for patients [6–8].

Digital interventions hold great promise for improving health, with several studies highlighting the positive impact of phone-based interventions on treatment outcomes for a variety of diseases [9–11]. In 2017, the WHO conditionally recommended the use of digital technologies, such as video-based observation of treatment (vDOT) for drug-susceptible TB where the technology is available and can feasibly be implemented [12]. This approach allows patients to record themselves whilst ingesting the treatment and to share the video with a health worker without the need for any face-to-face interactions. These guidelines were extended in 2020 to include MDR-TB, while acknowledging the very low certainty of evidence and calling for additional studies to further investigate its effectiveness for TB treatment and elucidate considerations for its implementation in low and middle-income countries [13].

In March 2021, Médecins sans Frontières (MSF), in collaboration with the Ministry of Health (MoH), undertook a study to determine the feasibility of routine provision of an all-oral short course (OSC) treatment regimen for MDR-TB in the public health care sector of Eswatini. In the study, patients were either assigned to DOT by a health worker during daily visits to a health facility, or by a community treatment supporter (CTS) at their home, following standardised eligibility criteria [7]. In brief, patients who lived close to the facility, who had the means and ability to undertake daily facility visits were assigned health worker DOT, while those who could not attend the facility or preferred home visits were assigned a CTS. However, before implementation of the study commenced, a surge in Covid19 cases occurred, bringing the now well-documented disruptions to health services, including those for TB, across the southern African region [14–16]. In an effort to minimise Covid19 spread and in light of existing concerns about TB transmission risks to health workers and CTS, patients were provided with smartphones with a video function to enable vDOT to replace face-to-face DOT where possible.

Within the context of the oral short course treatment feasibility study [17], we embedded qualitative research that aimed to describe the experiences of people living with MDR-TB, healthcare workers and their family members with vDOT, with a view to understanding how it could support adherence to MDR-TB drugs.

## Methods

### Study setting

The Shiselweni region of Eswatini has a population of 210,000 and the highest HIV prevalence in the country, at an estimated 26.5% among adults aged 15 years and older [18]. MSF has been supporting the Ministry of Health to deliver HIV and TB services in the region since 2007, including the national TB referral ward in the Nhlangano health center, which provides TB screening services for patients and their contacts as well as tuberculosis preventive treatment. In 2022, 96 drug-resistant MDR-TB patients were treated in the ward. MSF also follows a community outreach TB support approach for the predominantly rural Matsanjeni and Hlathikhulu zones located in this region. This outreach programme supports the TB ward with both passive and active TB case finding.

### v-DOT implementation

Introduction of vDOT in this context has been described in detail previously [17]. In brief, vDOT was implemented using the SureAdhere application [19] which had previously been used for monitoring drug-sensitive TB care and was adapted for this study to allow vDOT for patients receiving DRTB treatment. Eligibility for vDOT was defined as patients aged 18 years and over, receiving treatment for DRTB in the absence of clinical symptoms of fever and pulmonary decompensation. Additional eligibility criteria included interest from the patient in opting for vDOT and an assessment from the study nurse that the patient was able to operate the application. Eligible patients initiated VDOT at the time of treatment initiation and received a practical introduction to video recording and signed a consent form. The typical duration of the videos was under two minutes, being sufficient for the patient to mention which medications were being taken and to report any side effects being experienced or any other concerns associated with treatment-taking. The patient then videoed the consumption of the drugs including showing their empty mouth to demonstrate that the drugs had been swallowed. vDOT used in this context was asynchronous as videos could be taken reviewed by nurses after they had been uploaded by the patients, rather than requiring a real-time connection.

Several mechanisms were in place to ensure security of the patient data. The videos recorded by patients were automatically encrypted by the application, time-stamped and uploaded to a secure cloud-based server. Videos were automatically deleted from the phone once uploaded to avoid patient privacy breaches should smart phones be lost. Trained health workers could only view videos through a password-protected secure interface located in a private office.

Adherence to TB treatment was documented by the health workers into paper-based registers which were subsequently entered into the national electronic database. Patients who did not submit a video were sent a reminder by WhatsApp, and then further followed up by a phone call if the video was still not submitted. In the event of non-response, a home visit was organized.

### Sampling and recruitment

Twenty patients aged 20–64 years old were enrolled in the wider study to pilot the MDR-TB oral short course treatment at Nhlangano health center, Hlatikhulu government hospital and Matsanjeni health center. Enrolled patients were also eligible to participate in the qualitative study if they were sufficiently well enough to be interviewed. Among the twenty patients in the wider study, nineteen patients (six female and thirteen male) were eligible and invited to participate in the qualitative study, while one male patient considered too unwell to be interviewed and thus deemed ineligible. Eligible participants were provided with information about the qualitative study by a health worker. Those who expressed an interest in participating were then approached by a member of the qualitative research team via telephone or face-to-face, depending on their preference, to complete study enrolment procedures. Of those who were invited, one woman with MDR-TB declined to participate. Among those who agreed to participate, ten patients (56%) had chosen to use vDOT for treatment support, while 8 (44%) had opted for the CTS model.

Following their recruitment, each participant was asked whether they would be willing to invite a caregiver to participate in the study. Overall, nine of the 18 participants agreed to a caregiver or CTS being approached (seven patients using vDOT and two with CTS), and all nine who were approached agreed to participate in the study.

The qualitative team purposively sampled healthcare workers to participate in the study, ensuring a range of cadres (doctors, nurses, psychologists) among those implementing MDR-TB services at Nhlangano, Hlathikhulu and Matsanjeni health facilities. Among the ten healthcare workers who were approached, all ten agreed to participate in the study.

Appendix 1 presents the characteristics of all study participants.

### Data generation

#### People living with MDR TB

Once recruited for the qualitative study, patients who had been assigned to a CTS for adherence support were contacted to fix a date for their first in-person in-depth interview – aided by a topic guide developed for this study – at a time of their convenience at their home, approximately one month after initiation on treatment. The first interview covered their experiences and understandings of receiving an MDR-TB diagnosis, receiving OSC TB treatment, CTS-provided DOT, as well as issues around living with MDR-TB and their daily routines. A second interview was conducted with these participants after culture conversion, approximately four to six months later. The second interview focused on how their experiences and attitudes evolved over time. Interviews lasted approximately 45 to 60 min.

For participants who had been assigned to vDOT, data were generated through the smartphone chat application, WhatsApp. In this case, participants were contacted once a week by the researcher on WhatsApp and prompted to provide accounts of their experiences of using vDOT, in addition to their experiences with the TB diagnosis, adherence to MDR-TB drugs and any issues around living with TB infection. All possible formats of data on WhatsApp were encouraged, including text, video, photo, and audio recordings. WhatsApp contacts occurred at least once a week until no new issues emerged from the exchanges.

Healthcare workers were interviewed once, at least two months after the start of the study. The topic guides covered their experiences with diagnosing, treating, and counselling MDR-TB patients as well as their attitudes and perceptions about patient treatment support via CTS and vDOT.

Caregivers were also interviewed once, approximately two months after their relative (or patient under their care for CTS), had started treatment. The interviews explored their experiences with caregiving and perceptions towards providing treatment adherence support or towards vDOT.

Most in-depth interviews were conducted in Siswati, while interviews with healthcare workers were in either Siswati or English or a mix of both, according to the respondent’s preference. WhatsApp exchanges were in Siswati. All interviews were audio-recorded, and WhatsApp exchanges were typed out into Word documents, in both cases with the study participants’ consent. Interviews lasted approximately sixty minutes on average. To ensure safe data storage and protection, the audio-recordings were uploaded to a server, only accessible by staff in the qualitative research department. This was followed by translation of the interview into English during the transcription process, with these data also stored and accessed through the secured drive.

### Data analysis

Data were analysed thematically by reading through the data and identifying patterns in meaning which were then captured under codes and subsequently arranged to derive themes. Open coding was initially used to explore and consider all possible meanings within participant accounts that aligned to the study aim. Following this open, descriptive coding, the data were further reviewed, moving between concrete data and abstract concepts, raising the analysis to an interpretive and conceptual level [11, 20, 21] The coding scheme was regularly revised according to concepts emerging from the data. Analysis involved connecting emergent findings from the WhatsApp exchanges and IDIs including exploring how these may confer or differ. [22]. NVivo11 was used to aid coding, with analytic memos to track the development of the analytic process.

### Ethical considerations

Ethical clearance was obtained from both the MSF ethical review board and the Eswatini health and human research review board (EHHRRB) under ID numbers 2037 and SHR 250/2020 respectively.

## Results

Four themes emerged from the data that captured experiences with CTS-provided DOT and vDOT: stigma, efficiency, perceived risks of TB acquisition and patient autonomy.

### vDOT as a better tool to manage TB-related stigma: *“No one can gossip”*

Many participants who used vDOT said they appreciated the privacy, given that only the patient and the nurse viewing the video were involved. This level of privacy was perceived to shield them from stigmatising attitudes and behaviours that they may otherwise have been subjected to by persons observing them visiting the health facility or being visited by a CTS to facilitate daily pill-taking. As one man explained:

It’s so good for me…No one can gossip or spread what’s going on with me, as this is a secret between me, the nurse, and the phone…. provided they are able to see at the hospital each day that I take my treatment (Male patient, vDOT).

However, the privacy afforded to patients through vDOT could also be removed by healthcare workers if they were perceived to not be adhering to their treatment (at least by not submitting their daily videos). One patient recounted how his TB status was disclosed when a health worker came to remind him about uploading his daily videos, as his medication was then made visible to other people in his neighbourhood:

There was this one who came here to talk to me about not uploading the videos. They came and harassed me, so I was annoyed. … They even came with my pill re-fill but with the pills hanging, seen by everyone here. Hey! I was hurt that this person did not respect me, for me to respect her too. (Male patient, vDOT)

### vDOT as a more efficient tool: ‘Now I can monitor several patients at the same time’

A regularly cited key benefit of vDOT from patients was that it saved time and resources, as well as affording them greater flexibility. This was mirrored in the accounts of some healthcare workers who found that vDOT simplified their work since they were able to monitor patients’ treatment journeys through a video, without the need to physically move to the patient’s home (as had been the case when patients’ movements were limited by the Covid19 epidemic) or vice versa:

It was tiring moving from one home to another and some of them are really far. Now I can monitor several patients at the same time without having to travel every day (HCW).

However, on the other hand, some health workers challenged the notion that vDOT lightened their workload as they noted that watching the videos was time-consuming and competed with the need to provide consultations to other patients who were waiting in person:

The challenge is that it would be too much work to view many videos and have to attend to patients waiting outside (HCW).

vDOT was perceived by healthcare workers to be adequate for assessing the health condition of the patient and determining whether they required hospitalisation or not. On the other hand, vDOT, especially over the weekends, could sometimes lead to delays in addressing identified needs for treatment or psychological support, since the health worker or caregiver was not on hand to provide it. Indeed, the consistent availability of the community treatment supporter was identified by some patients as an important advantage and reassured them that their needs would be met:

He [the CTS] is always available, he is not going anywhere, yes. (Male Patient, CTS-DOT).

In addition, vDOT was viewed by some caregivers as providing the best link between the patient and a facility-based health worker, because it enabled them to discuss their treatment-related issues from the comfort of their homes, with minimum effort and finances.

### vDOT protects… “it is good at infection control”

Some health workers were grateful for vDOT because it reduced their contact with patients which in turn reduced their risk of exposure to TB infection:

The good thing about this video thing is that I do not have to see many patients physically all the time. I sit in my office and monitor them from here, which helps me avoid getting sick because of seeing many patients with TB. (HCW)

Other health workers pointed to the additional benefits of vDOT as reducing their exposure to Covid19 which was prevalent at the time of the study:

It is good at infection control especially during this COVID era because not many patients come to the health center since they are using videos, and we can use the videos to check whether they are taking the treatment (HCW).

While HCWs appreciated that VDOT minimized their exposure to infection, some family members did not perceive themselves as being at risk of infection from their patients whose treatment adherence was monitored via VDOT.

I heard that we must be careful about how we sit [together] as it [TB] is more like the current disease [Covid-19] but then we were warned not to discriminate him, and I don’t do that, even now if he were here, he would be seated next to me (Family member.

### vDOT promotes patient autonomy: “it helps take the load away from us”

Several patients who preferred the vDOT option acknowledged that the requirement to submit daily videos served as an additional incentive to treatment adherence, while others indicated that provided them with a greater sense of autonomy and motivated them to stay on track as they strived to complete treatment in a more independent way:

Dude this [vDOT] is the best way of continuing to take the treatment on my own. (Female patient, vDOT)

In addition, vDOT was perceived as enabling patient accountability to healthcare workers by providing an easy way to provide “proof” of their adherence.

I am glad that there is actually someone always watching me because if I were alone, they would be saying, aw, this person is not taking the pills. (Male patient, vDOT)

Indeed, some healthcare workers indicated that their preference for vDOT stemmed from the ability to directly witness the treatment adherence of their patients on video, rather than having to rely on reports of adherence from lay health workers which they did not always trust.

Some CTS just side with patients when they cry about the pill burden. Sometimes the CTS just gives in and does not report that a patient is missing some doses. (HCW)

However, not all patients felt comfortable with using the vDOT technology, with some found the idea of recording themselves ingesting their drugs off-putting, ultimately preferring a face-to-face intervention over the video submissions:

*[…] it makes you look like a cartoon! (Male patient*,* vDOT)*

Some family members felt somewhat excluded from caring for family members who had been assigned to vDOT, with some feeling that it rendered them redundant in as far as providing treatment support for their relatives. However, most family members appreciated vDOT because they felt that it saved them from some of the emotional burden associated with managing their sick relatives, especially those who became agitated when taking the medication, and enabled them to quickly request support when needed:

I actually see that it does play a role because if there is anything that he is running short of, or missing, he calls. ‘Hey! Such and such a thing is not here (Family member).

Several family members felt that vDOT or CTS-provided DOT had the potential to relieve them from the burden of ensuring patients in their care adhered to treatment, because the responsibility of daily treatment-taking lay with the patient and the healthcare worker or their community treatment supporter.

When he is taking the pills, we usually find him putting everything together, and we also see him there on the phone, and then we say eh, maybe we need to leave you so that we do not disturb you (Family member).

…this thing of having someone to assist is good, it might be an expense to someone, but it helps take the load away from us and looks like it is effective, you see? (Family member)

In order to promote patient’s ability to autonomously record and submit the videos, smartphones were provided, along with torches to ensure videos were recorded with sufficient light. Some family members expressed surprise that patients were given these items:

[…] yes, I am surprised that now people get sick and are given things like this. First it was the phone and then the globes (torches)! (Family member)

While the items were generally appreciated, they could also on occasion be a source of jealousy, and the need for the capacity for charging such devices was also noted.

## Discussion

This qualitative study explored experiences with vDOT for people living with MDR-TB taking oral short course treatment as well as the views of their caregivers and health workers, in rural Eswatini. Few qualitative studies have reported on experience of vDOT in African settings for people living with TB, and this is the first to our knowledge among people with MDR-TB. Our findings suggest that many participants with MDR-TB appreciated the greater privacy, reduced time and cost burden, and greater treatment autonomy associated with vDOT, while health workers appreciated the reduced exposure to MDR-TB infection that this adherence model afforded to them. Most family members appreciated vDOT, although some resented feeling removed from the process of supporting loved ones.

Our results showed that greater privacy over treatment-taking enabled patients using vDOT to be less exposed to TB-related stigmatisation, by making their disease status less visible to others in the community. These findings support the existing limited evidence base that vDOT can reduce levels of stigmatisation among TB patients [23], thereby addressing one of the key barriers to adherence [24]. Furthermore, vDOT was seen to promote patients’ autonomy and independence in their treatment journey, whilst still enabling them to access psychosocial support, which has been documented as an essential element of adherence in any studies. [25, 26].

Our findings suggest that vDOT was considered more convenient by many healthcare workers and people living with MDR-TB because it was time-saving, consistent with findings reported elsewhere [27]. However, in our study, healthcare workers’ concerns that time to watch videos and respond to certain issues raised by the patients could add to their workload and could increase waiting times for other patients in the facility need to be addressed by including video viewing time in their rosters, or task-shifting video reviews to trained lay cadres, with supervision by nurses. Furthermore, whilst vDOT may reduce the costs for lay workers associated with undertaking daily visits to patients in their homes, it has still been evaluated as more expensive compared to DOT provided by CTS or family members, largely due to the up-front technology expenditure [28]. Following the successful implementation of this study, the use of vDOT was scaled up nationally in Eswatini by the Ministry of Health. However, to minimise costs, it was provided to patients who had access to smartphones [17]. Further studies to investigate the relative costs and cost-effectiveness of a mix of vDOT and CTS-DOT interventions that could support patient preferences in this setting would provide valuable information for policymakers considering its scale up.

While other studies have shown that some people with TB find it challenging to find sufficient privacy in their homes to record their videos, or were concerned about being ridiculed by family members for their recordings, these concerns were not observed in our setting [29]. Similarly, we did not hear any reports of participants being concerned about the potential security breach of videos being shared online, which was noted in other settings, perhaps due to the confidence with security mechanisms with the application [29]. Other studies exploring experiences with vDOT have found that challenges with technology and electricity have proved problematic for effective use of this adherence [29]. In our study, smartphones were provided to participants, along with airtime, and a torch to enable the videos to be made. While this addressed some of the technology-related challenges experienced in other settings, the additional provision of equipment to participants would increase the cost of the intervention.

Furthermore, despite the strengths of vDOT, many participants appreciated in-person visits by CTS and seemed more likely to adhere to infection prevention and control measures given the daily in-person patient supervision which corresponds to findings from a similar study in Cambodia [30]. Previous research from the same setting in Eswatini demonstrated that the CTS model for adherence support is also widely considered acceptable by patients and family members [31]. In view of the finding that some participants struggled with using smartphones, or did not appreciate the vDOT approach, our study underlines the importance of providing patients with choices regarding adherence support options to ensure that the provided modality is the best suited for the patient’s specific needs. The provision of a choice of options, along with clearly presented information on the relative pros and cons of each is one of the principles at the heart of patient-centred care and can lead to better levels of engagement with health interventions and better health outcomes [32]. Further support for the provision of options comes from previously reported findings from this setting that about half of all DRTB patients enrolled in the oral short course treatment feasibility study opted for vDOT and demonstrated similarly favourable treatment outcomes when compared to those receiving in-person DOT [17].

Other reported advantages of vDOT in our study were that it limited exposure to MDR-TB among healthcare workers (as well as limiting exposure to Covid19 for health workers and people with TB patients during this study). Several studies from southern Africa have indicated a substantially higher burden of active and latent TB, including drug-resistant TB, in healthcare workers compared to the surrounding community or general population [33, 34], suggesting that the vDOT intervention could play an important role in preventing some of this transmission.

Our findings should be considered in light of various limitations. Firstly, MDR-TB care provided through services supported by MSF may not be generalisable to other settings in the country, leading to different patient and health worker experiences. Furthermore, patients from this rural area of Eswatini may differ from those in other regions in terms of their familiarity with mobile devices and prior exposure to research studies, which requires caution in terms of assuming representativeness of the sample.

One of the strengths of the study was the novel use of the WhatsApp application to generate data among patients with vDOT, which allowed the researchers to gain insights into participants’ experiences from a variety of locations and at several time-points, without the need for frequent face-to-face contacts for interviews, and at a time that was most convenient and spontaneous for the participants. While typed responses of WhatsApp could be more succinct than those provided in in-depth interviews, the use of audio recordings on WhatsApp enabled patients to vocalise their answers to some questions.

Although other studies have also shown that smartphone applications can enable greater access to participants who may be traditionally hard to reach, may reduce infection transmission risks, and allow for data to be accessed in real-time, further evaluations of these applications for generating data should be encouraged in order to extend the evidence base further [35].

## Conclusions

This study provides additional evidence to support the WHO conditional recommendation for the use of vDOT among patients with MDR-TB. In this setting, vDOT was generally appreciated by MDR-TB patients, their family members and health workers as it addressed barriers to adherence which could contribute to improved treatment completion rates and also reduced workplace exposure. However, patients should be offered an alternative to vDOT where this modality does not suit their circumstances or preferences.

### Electronic supplementary material

Below is the link to the electronic supplementary material.

Supplementary Material 1

## Data Availability

Datasets used for analysis may be made available upon formal request through the corresponding author.

## Appendix

**Appendix 1:** Characteristics of each participant group and data generation methods used

Age-group	Gender		Data generation method		
Patients	Male	Female	SGD	IDI	
20–35	3	4	3	4	7
36–50	4	1	4	1	5
> 50	5	1	2	4	6
	**13**	**5**	**10**	**8**	**Total** =**18**
**HCWs**					
31–40	2	6	0	8	8
41–50	0	2	0	2	2
	2	8	0	10	**Total** =**10**
**Caregivers**					
20–35	2	0	0	2	**2**
36–50	0	2	0	2	**2**
> 50	1	4	0	5	**5**
	**3**	**6**	**0**	**9**	**Total =9**
Spouse	Mother	Sibling	Cousin	CTS	**Relationship to patient**
**2**	**2**	**2**	**1**	**2**	**Total Participants = 37**

*Overall number of participants enrolled in the study = 37

Key

SGD Smartphone generated diary

IDI In-depth interview

HCW Healthcare worker

CTS Community Treatment Supporter
