# Supplementary material for: Understanding the role of video direct observed therapy for patients on an oral short-course regimen for multi-drug resistant tuberculosis: findings from a qualitative study in Eswatini
Source: BMC Infect Dis. 2024 Aug 15;24:829. doi: 10.1186/s12879-024-09744-9 (PMC11325818; doi:10.1186/s12879-024-09744-9)
Supplement: Supplementary file 1 — Supplementary Material 1 [file 12879_2024_9744_MOESM1_ESM.docx]

**Annex 12: Prompts SGD**

This list is summarizing some ideas of what to ask patients using OSCT and VOT about their experiences. It should help to make sure we frame the information we want from the patients right.

We expect the participants to share their experiences as instant text messages, voice messages, pictures, videos. The prompts will evolve over time and be adapted to the participants. New prompts will be included.

1. Tell us about your daily life? How does your life look like?

2. How do you feel today? What has changed since you are on treatment? Any challenges with the treatment in the last week?

3. How would you describe your symptoms since beginning the treatment?

Are you getting better/worse/same? Any new symptoms? Which symptoms better/worse/gone?

4. How would you describe any side effects caused by the treatment? Do you experience them?

5. Do you find it easy to be on OSCT for DR-TB? Why? Why not?

- Does the usage of VOT make you more independent?

6. Tell me how you feel about VOT? What do you like about it? What do you dislike about it? How has VOT changed your life?

7. Are there any people supporting you with the treatment? Can you tell me about them?

8. How would you describe the VOT? What could improve? What do you like about it?

- What are the most important tools for you during this treatment? What should be improved?

9. Do you find VOT easy or difficult to use? Can you tell me why?

**Annex 13: Interview guideline IDIs patients**

| This topic guide provides a broad outline of questions to ask, but these should be adapted to the account given by the participant. Not all questions are relevant to ask all participants, adapt and skip as appropriate.  Through the course of the interview, we are trying to build a picture of patients’ experiences of receiving an MDRTB diagnosis, their experience with OSCT and their feelings towards VOT. We want to understand the patients’ responses and processes of coping with diagnosis and treatment and the acceptability of VOT in this context.  An in-depth interview is not like a questionnaire, it should be led by the participant’s account and have open-ended questions, where we encourage participants to tell their stories rather than “yes”/”no” answers. Suggested probes are listed to encourage conversation.  **Part 1: Introduction**  The aim of the first part of the interview is to develop a rapport with the participant you are  interviewing. It is important to develop a non-judgemental tone throughout the interview and to convey that there are no right or wrong answers.  Start by making sure that the participant is comfortable and at ease, and then provide a recap about the study.  Turn digital recorder on. |
| --- |

Example introduction – adapt as appropriate.

Thank you for agreeing to talk to me today. As I explained earlier, we are studying the perspectives and experiences of patients with DR-TB on OSCT. We are researchers, not doctors or nurses.

However, we have been asked to assist the health services by evaluating the treatment and counselling of AHI so that implementation of these services can potentially be improved in the future.

We are interested in your opinion today; everything you say is very interesting to us. I will not talk much, but I want you to talk freely, and as much as you want. There are no “good” or “bad” answers.

1. Can you tell me a bit about yourself?

Probes: Where you are from? Where do you live now? Age? What you do for a living?

2. Can you tell me about your family life?

Probes: Who do you live with? Relationship status – probe different partners, children/how many children?

3. Can you tell me what you do on a typical day?

Probes: What do you do from you get up in the morning till you go to bed?

| **Part 2: Circumstances around the TB infection**  The aim of this part of the interview is to gain an understanding of the participant’s perceptions of their TB risk and possible exposures prior to their diagnosis. We would like to understand the circumstances under which the participant may have been infected. |
| --- |

I would like to ask you some questions about before you received an MDRTB diagnosis – how you perceived your risk of getting infected and how you protected yourself.

4. Before you were told that you had DR-TB, did you ever feel that you were at risk of getting TB?

Probes: Was getting TB something you worried about? Why/why not? Household members with TB?

5. Can you tell me about any steps that you took to protect yourself from getting infected with TB?

6. Did you ever have any discussions with any health professional about your risk of getting infected with TB?

Probes: Why/why not? Who? What did you discuss?

| **Part 3: Experience with receiving the diagnosis of DR-TB**  The aim of this part of the interview is to gain an understanding of the experiences of the patient receiving an DRTB diagnosis - the patient’s account of what happened at the health centre and the patient’s response and process of coping with the diagnosis |
| --- |

I would like to know what you understand about DR-TB and your experiences receiving your

diagnosis.

7. Can you please explain to me the reason(s) you came to the health centre on the day that you found out that you had DR-TB?

Probes: encouraged by friend/family/partner, well but worried about a risk, came for another unrelated service (or ANC etc), partner tested/was sick/died, symptom(s) you had experienced? For how long? What did you think it was?

8. Can you please describe to me everything that happened at the health centre when you tested?

Probes: Who was there? What did they ask you? Which/how many tests were taken? Which

information did you receive? How long did it take – same day/days?

9. Can you tell me about how you felt when you were told that you have TB?

Probes: What went through your mind? How did you feel? What did you do?

10. Can you tell me about how you felt when you were told that you had DR-TB?

Probes: What went through your mind? How did you feel? What did you do?

Was everything clear to you at the health centre – the diagnosis/information, you received?

Probes: Was there anything that you were unsure about? Questions/doubts that were not answered?

12. What happened after you received the result?

Probes: Did you talk to anyone about it? Who? What was useful/not useful?

13. In your opinion, could anything have been done differently at the health centre in order to make it easier for you?

Probes: Counselling, the time you spent/waited for the results, information etc.

14. Can you please explain to me in your own words what you think DR-TB is and how it is different from a “normal” TB?

| **Part 4: Facilitators and barriers to OSCT initiation and difficulties in family live and opting out on VOT.**  The aim of this part of the interview is to gain an understanding of what drives or hinders linkage to treatment. Furthermore, we would like to know how the OSCT treatment is experienced. The questions should be adapted to the specific participant; depending on their individual circumstances |
| --- |

I would like to know about your experiences with OSCT and potential changes/ challenges in family life.

15. Can you tell me about whether you have been offered OSCT following your diagnosis?

Probes: when and where offered, by whom?

16. Why did you start OSCT – and when?

Probes: Same day or later? If given pills on same day, actually consumed them? Or waited?

17. Why did you decide to start OSCT?

Probe Did you feel pressured to start treatment?

18. How do you feel about the treatment?

Probes: Side effects? Difficulties? Benefits?

19. Who/ what assists you with your treatment?

Probes: What has worked well?

20. How is the interaction with the HCWs?

Probes: What was/is good/not good? Did you have all your doubts and questions answered? Were your concerns listened to?

21. Any suggestions on how to improve the treatment?

Probes: Probe for reasons.

| **Part 5: Everyday experience of OSCT for DRTB**  In this part of the interview, we are trying to understand how the everyday life of the participant has been influenced by the diagnosis. We specifically want to explore what might have changed in the participant’s life and how the participant experience being on OSCT, including potential challenges. |
| --- |

I would like to ask you some questions about your everyday experience with OSCT.

22. I’d like to know everything that happened in the weeks after you were diagnosed with DR-TB? Did anything change in your life since then?

Probes: Changes in social life? Something you do, which you did not do before/something you did before that you do not do now? Challenges/concerns?

23. What was the adherence support approach that you were offered? Can you describe VOT to me?

24. Can you tell me some of the reasons why you deciding against VOT?

Probes: Challenges with usage of technology? Mistrust in technology? Network coverage?

25. Anything that could have helped you to opt for VOT?

| **Part 6: Wrapping up.**  Before closing the interview, give the participant an opportunity to make any further comments about the topics discussed or to ask questions.  Thank the participant for his/her time and for sharing experiences and views. |
| --- |

Are there any issues we discussed today that you would like to talk more about? Do you have any comments or questions about the topics that we discussed today?

End of interview – thank the participant.

**Annex 14: Interview guideline IDIs HCWs**

This topic guide provides a broad outline of questions to ask, but these should be adapted to the account given by the participant.

Through the course of the interview, we are trying to build a picture of health practitioners’ experience and perception of diagnosing, treating, and counselling patients with AHI – the strengths and weaknesses of the current implementation. For example, what are the difficulties that the health workers face themselves diagnosing, treating, and counselling patients with OSCT DR-TB patients and with the usage of VOT? How well equipped do they feel in terms of training that they have received to deliver this new approach?

An in-depth interview is not like a questionnaire, it should be led by the participant’s account and have open-ended questions, where we encourage participants to tell their stories rather than “yes”/”no” answers. Suggested probes are listed to encourage conversation.

| **Part 1: Introduction**  The aim of the first part of the interview is to develop a rapport with the participant you are interviewing. It is important to develop a non-judgemental tone throughout the interview and to convey that there are no right or wrong answers.  Start by making sure that the participant is comfortable and at ease, and then provide a recap about the study.  Turn digital recorder on. |
| --- |

Example introduction – adapt as appropriate.

Thank you for agreeing to talk to me today. As I explained earlier, we are studying the experiences and perceptions of health workers involved in the diagnosis and treatment of DR-TB patients on OSCT using VOT. We are inviting you to be interviewed to discuss your experiences with this approach.

The aim is for us to better understand your experiences and the problems you might face with this implementation so that it could potentially be improved in the future.

We are interested in your opinion today; everything you say is very interesting to us. I will not talk much, but I want you to talk freely, and as much as you want. There are no “good” or “bad” answers.

Can you tell me about yourself?

Probes: Where you are from? Age? Your position here? How long working in this role?At this health centre?

Can you describe a typical workday to me?

Probes: How many patients do you usually see in one day? How long do you spend with the patients?

What is the first thing that you do? And then what do you do?

What do you like most about your job?

Probes: Specific tasks? The purpose of the job? Role?

What do you find most difficult about your job?

Probes: Specific tasks? The purpose of the job? Role?

| **Part 2: Experiences with diagnosing, treating, and counselling patients with DRTB on OSCT**  The aim of this part of the interview is to understand health practitioners’ experiences with diagnosing, treating, and counselling patients. For example, what difficulties the health practitioners face themselves, and how they support patients when they face challenges especially in relation to linkage to treatment and treatment support. |
| --- |

Can you tell me specifically about the work that you do in relation to patients with DR-TB on OSCT?

Probes: What is your role? What activities does it involve?

Can you tell me about how you explain TB to patients?

Probes: What is difficult? What are the most important things to explain? How easy/difficult is it for the patient to understand the diagnosis?

In your experience, what are some of the concerns that might mean that DR-TB patients do not adhere well to OSCT?

How do you explain the treatment and how do you support the treatment?

| **Part 3: VOT** |
| --- |

How does the VOT work for you? What do you think about digital health services?

Probes: Any challenges? Benefits? Drawbacks?

Do you think that VOT improves or undermines treatment outcomes?

Probes: How? Why not?

How do patients react to the offer of the digital health component/ VOT?

Probes: Is it easy or difficult to understand and use for them?

What needs to improve in the VOT?

Probes: Anything missing?

| **Part 4: Health practitioners’ training and support**  The aim of this part of the interview is to understand how well-equipped health practitioners feel in terms of training and support to diagnose, treat, and counsel patients. |
| --- |

Now, I would like to ask you some questions about potential challenges diagnosing, treating and counselling patients.

Can you tell me about any challenges that you or your colleagues face when diagnosing/treating/counselling patients with DR-TB on OSCT?

Probes: Any frustrations?

Do you feel you have the adequate training and techniques to work with patients part of the study?

Probes: Please elaborate? Some areas where you would like more training/techniques?

Do you feel you have adequate support?

Probes: Please elaborate? Some areas where you would like more support?

How does working with DR-TB patients on OSCT and VOT fit with your other daily activities and tasks?

Probes: Do you feel you have adequate time? How has your role changed since the implementation started? Has it had an impact on the rest of your tasks?

Can you think of any ways in which things could be better organized, which would make it easier for staff to work in the study?

Probes: Please elaborate

What do you think about the potential for a national roll-out of this new approach?

Probes: Feasibility? What would be the benefits/challenges?

What do you think should be considered or addressed before?

Probes: Something that does not work now, that should be changed?

| **Part 5: Wrapping up.**  Before closing the interview, give the participant an opportunity to make any further comments about the topics discussed or to ask questions.  Thank the participant for his/her time and for sharing experiences and views. |
| --- |
